# Chemical Physiology

**Published:** 1902-09-27

**Authors:** 


### CHEMICAL PHYSIOLOGY.

The importance and the far-reaching influence of bio-chemical investigations were well shown in the remarkable address given by Professor Halliburton before the British Association at Belfast. Not unnaturally the early efforts of the physiological chemists have concerned themselves chiefly with the composition of the blood, which is an easily accessible fluid, and with problems touching upon digestion which of all physiological processes seems the most purely chemical. But the light which is being thrown upon the composition of the blood in various conditions, upon the nature and causes of its various reactions, upon the chemical constitution of the bacteriolysins and antitoxins found therein, and of the "immune body" and its "complement" of which each of these substances is shown to consist, all tends to illuminate many dark fields having to do with the nutrition of the tissues whether in health or in disease, while the investigations which have lately been made in regard to the varying nature of the digestive secretions seem to suggest that much which has hitherto been regarded as due to reflex action is really a question of biological chemistry, the varying results being due to variations in the chemical stimuli present at different times. The extent and variety of the lesions set up by the ingestion of a gross poison such as arsenic have lately given startling evidence of the degree to which degenerations of tissues may be due to well recognisable chemical poisoning; and the investigations which have given a chemical value to the toxins found in certain diseases have put many of the pathological changes found in these diseases into the same category. Thus, both in physiology and pathology, the progress of the immediate future seems to depend greatly upon the biological chemist, by whose labours we hope to ascertain the nature of those more intimate protoplasmic changes which are concerned in nutrition, whether that be the normal nutrition which is health, or the abnormal which leads to disease. All of this is of supreme interest to medicine; for if it be true that morbid nutrition is a mere matter of chemistry, a thing to be altered and set right by the addition or subtraction of a molecule, and if it be true that febrile diseases represent a contest between microbes producing poisons and cells producing antidotes—chemical products capable of analysis and in the future it is to be hoped capable of neutralisation—a time must come when we shall not have to stand by with folded hands but shall be able to intervene with a confidence born of knowledge.

